# Genome Sequences of Eight *Aspergillus flavus* spp. and One *A. parasiticus* sp., Isolated from Peanut Seeds in Georgia

**DOI:** 10.1128/genomeA.00278-16

**Published:** 2016-04-14

**Authors:** Paola C. Faustinelli, Xinye Monica Wang, Edwin R. Palencia, Renée S. Arias

**Affiliations:** National Peanut Research Laboratory, Dawson, Georgia, USA

## Abstract

*Aspergillus flavus* and *A. parasiticus* fungi produce carcinogenic mycotoxins in peanut seeds, causing considerable impact on both human health and the economy. Here, we report nine genome sequences of *Aspergillus* spp., isolated from Georgia peanut seeds in 2014. The information obtained will lead to further biodiversity studies that are essential for developing control strategies.

## GENOME ANNOUNCEMENT

The United States is the third largest peanut producer in the world, with Georgia yielding 49% of the national total (1). People in the United States consume an annual average of 6 lb of peanut products per capita (2). The fungal *Aspergillus flavus* and *A. parasiticus* spp. typically produce aflatoxins, which are considered the most powerful mycotoxins associated with liver cancer, child growth impairment, and acute toxicoses (3–5). These two members of the section *Flavi* contaminate agricultural products and, consequently, foodstuffs, including peanut products (6). Stringent regulations have been imposed on the level of aflatoxins allowed for marketed peanut products (7). The impact of aflatoxins costs the peanut industry between $25 and $58 million annually in the United States (8, 9). Despite all efforts, more than 5 billion people worldwide are at risk of exposure to aflatoxin (10). Currently, there is a need to develop systemic and effective approaches to manage aflatoxin contamination of susceptible crops.

The natural populations of *Aspergillus flavus* and *A. parasiticus* are diverse (11), and since evidence of sexual reproduction was found for these species (12, 13), genetic variability is expected, especially in the aflatoxin biosynthesis gene cluster (AB cluster) (4). Although the sequences of the genes and the intergenic distances in the AB cluster are considered well conserved (14), deletions are common and seem to have different patterns that may be due to processes of adaptation/evolution in nature (15, 16). Here, we present the genome sequences of eight isolates of *A. flavus*, three of which are nonaflatoxigenic, and one isolate of *A. parasiticus*. The DNA sequences obtained will provide valuable molecular information to determine genetic diversity in the section *Flavi*—data necessary to select appropriate target genes to control aflatoxin accumulation in crops.

More than 240 *Aspergillus* spp. were isolated on MDRB medium (17) from peanut seeds from the state of Georgia, United States, during 2014, and the isolates were fingerprinted with 25 InDel markers (unpublished). Genomes of nine representative isolates from various clades in the cluster analysis were sequenced using next-generation sequencing (Illumina HiSeq2500) at the University of Washington, United States. Prior to assembly, the sequence reads were processed using CLC Genomics tools (CLC Genomics Workbench version 8.5.1, Qiagen, Denmark) to remove sequence adapters and trim off any ambiguous nucleotides. Using Geneious version 8.1.7 (18), the *A. flavus* processed reads were mapped to the published *A. flavus* NRRL3357 genome (19), and the *A. parasiticus* read was mapped to its corresponding published genome (20). The total length of each draft *A. flavus* genome ranged from 35.8 to 36.5 Mbp, slightly smaller than the *A. flavus* NRRL3357 genome (37 Mbp) (19), and the G+C average was 48.3% (Table 1). Alignments of the AB Cluster with the published *A. flavus* NRRL3357 cluster (19) using the Clone Manager tool (Clone Manager version, 9 Professional Edition, USA) indicated a 98 to 99% homology, except in *A. parasiticus*, which indicated an expected 81%.

**TABLE 1:** Listing of *Aspergillus* sp. isolate genomes released to NCBI

Isolate	Accession no.	*Aspergillus* sp.	Aflatoxin producer	Genome size (bp)	Fold coverage (×)	% G+C
26-3	LOAN00000000	*flavus*	Nonaflatoxigenic	36,329,774	38.9	48.2
54-2	LLET00000000	*flavus*	Nonaflatoxigenic	36,578,699	65.6	48.3
78-6	LOAO00000000	*flavus*	Nonaflatoxigenic	36,203,959	39.6	48.3
206-4	LOAM00000000	*flavus*	Aflatoxigenic	36,084,636	33.8	48.3
40-5	LIZI00000000	*flavus*	Aflatoxigenic	36,412,503	45.7	48.3
61-4	LIZJ00000000	*flavus*	Aflatoxigenic	36,322,355	39.3	48.3
72-5	LOAK00000000	*flavus*	Aflatoxigenic	35,993,818	29.8	48.3
79-2	LOAL00000000	*flavus*	Aflatoxigenic	35,835,174	32.9	48.3
68-5	LOAP00000000	*parasiticus*	Aflatoxigenic	30,136,366	36.9	48.3

### Nucleotide sequence accession numbers.

GenBank accession numbers for the nine genomes are listed in Table 1.
